# Early menopause and severity of rheumatoid arthritis in women older than 45 years

**DOI:** 10.1186/ar4021

**Published:** 2012-08-17

**Authors:** Mitra Pikwer, Jan-Åke Nilsson, Ulf Bergström, Lennart TH Jacobsson, Carl Turesson

**Affiliations:** 1 Department of Rheumatology, Institution of Clinical Sciences, Inga Marie Nilssons gata 32, 205 02, Malmö, Sweden; 2 Deptartment of Rheumatology & Inflammation Research, Institution of Medicin, University of Gothemburg, Guldhedsgatan 10 A, 405 30 Göteborg, Sweden

## Abstract

**Introduction:**

We aimed to investigate whether recognized hormonal predictors of rheumatoid arthritis (RA) also influence the severity of RA.

**Methods:**

One hundred thirty-four incident RA cases identified by four different local and national registers, who had participated in a community-based health survey between 1991 and 1996, were included. By a retrospective structured review of the medical records, information on the use of disease-modifying antirheumatic drugs (DMARDs), erosions on radiographs, rheumatoid factor (RF) status, and disability measured by using the health assessment questionnaire (HAQ) were collected. The variables were added to the SPSS TwoStep Cluster Analysis to reveal natural groupings of RA severity. Known hormonal predictors analyzed were breastfeeding history, history of oral contraceptive (OC) use, and menopausal age.

**Results:**

The mean age at RA diagnosis was 63.4 years; 72% were RF positive, and 28% had received biological treatment. Three clusters were identified, one with severe RA, one with mild/moderate RF-positive RA, and one with mild/moderate RF-negative RA. A significant difference (*P *= 0.005) was found in the distribution of clusters between patients with a history of early menopause compared with those with menopause after 45 years, with a higher proportion with mild/moderate RF-negative RA in the early-menopause subset. No major difference in severity of the disease was noted depending on OC use or history of breastfeeding.

**Conclusions:**

Early menopause was associated with a milder form of RA. Hormonal changes may influence pathways that are distinct from those leading to severe, progressive disease.

## Introduction

Rheumatoid arthritis (RA) is a chronic, inflammatory disease, with a female predominance [1,2]. Hormonal factors have been suggested to influence the risk of RA. A reduced incidence occurs during pregnancy, and also an increased rate of clinical remission among women with RA who become pregnant [3]. Low testosterone levels have been described in both women and men with RA [4-6], and a high incidence of RA is found during the peri- and postmenopausal period in women [7], suggesting that hormones and hormonal changes may influence pathogenesis and disease progression. The disease is heterogeneous and now often is regarded as a syndrome of several distinct phenotypes instead of being a single disease [8]. Hormonal factors could have diverse influences on older onset of RA compared with younger onset, as well as on seropositive and seronegative disease.

The effect of oral contraceptives (OCs) on RA has been the subject of many studies and meta-analyses [9-11], with diverging results. Variances in methods, doses, and types of OC, as well as population differences, have been proposed to explain some of these controversies. It has been suggested that OCs alter the course of RA, instead of acting as a predictor, and are associated with a milder type of disease and less disability [12,13]. In a previous study, we did not find any impact of a history of OC use on the risk of RA [2]. We did, however, demonstrate an association for early menopause (≤ 45 years) with increased risk of RA, and a protective effect of long-term breastfeeding [2,14] on postmenopausal onset of RA. Jorgensen *et al. *[13] reported that the mean duration of breastfeeding was longer in women who subsequently developed a severe RA phenotype. To our knowledge, no studies have been conducted on the influence of menopausal age on the long-term outcome of RA.

The aim of this study was to investigate whether hormonal factors, such as breastfeeding history, OC use, and menopausal age, influence the severity of RA.

## Materials and methods

### Malmö Diet and Cancer Study

The Malmö Diet and Cancer Study (MDCS) is a population-based prospective cohort study performed in Malmö, Sweden, between 1991 and 1996. Baseline examination included a self-administered questionnaire, anthropometric measuring, and blood sampling, stored in a biological bank. All individuals living in Malmö and aged 44 through 74, on 1 January 1991, were invited. In total, of 30,477 subjects (18,326 women) were recruited. Language problems and mental retardation were the only exclusion criteria [15].

By linking the MDCS to four registers (a community-based RA-register, the local outpatient clinic administrative register, the National Hospital Discharge Register, and the National Cause of Death Register), we identified individuals who had developed RA after their participation in the MDCS (≥ 1 year before RA diagnosis). Based on a review of the medical records, patients were classified according to the 1987 American College of Rheumatology (ACR) criteria for RA [16]. Female patients who been diagnosed before December 31, 2004, and fulfilled the ACR criteria from 1987, were included in the present study.

### Clinical-outcome measures

In a separate, structured review of all medical records, performed by one of the authors (MP), data were collected on clinical-outcome measures up to the date of the review (between February and October 2010) or to the last day of follow-up. Information on treatment with Disease Modifying Anti-Rheumatic Drugs, (DMARDs), including biologics, was collected. Treatment with biologics for RA in Sweden is to a great extent based on national evidence-based guidelines. These were first formulated in 2002 and were updated in 2004 and since 2008 on an annual basis [17]. Although changes in the guidelines occurred over the years, biologics are still recommended mainly for patients with severe RA that is refractory to traditional DMARD therapy [17]. Based on this, ever use of biologics can be used as a surrogate marker for severe disease. Data on exposure to biologics were also derived from linkage to a regional follow-up system for patients with rheumatologic diseases receiving such treatment, the South Swedish Arthritis Treatment Group (SSATG) register [18]. The SSATG was established in 1999 and includes patients from a total of 10 rheumatology centers, of which six are in the Region Skåne area in southern Sweden (~1.3 million inhabitants). The register has been compared with pharmaceutical sales data and found to cover more than 90% of anti-TNF-treated patients in the area [18]. Further clinical information collected from medical records included data on prednisolone treatment at diagnosis (or within 1 month before or after the time of diagnosis), severe extraarticular manifestations (pericarditis, pleuritis, vasculitis, Felty syndrome, glomerulonephritis, and interstitial lung disease) [19], and disability measured by using the Health Assessment Questionnaire (HAQ) [20] at diagnosis and after 1 year (the closest value within 6 to 18 months was accepted), 5 years (4 to 6 years accepted), and 10 years (8 to 12 years accepted) from diagnosis. The HAQ is a self-administered questionnaire, consisting of questions about of 20 activities of daily living in eight categories. This instrument is useful in monitoring the patient's course and response to therapy and in assessing functional disability. The HAQ form is distributed to all patients with RA before visiting the outpatient clinic at the University hospital in Malmö. The scale ranges from 0 to 3, with higher scores indicating greater disability. To be able to classify HAQ scores in relation to disease duration, the exact date of diagnosis was set as the day a rheumatologist gave a definitive (ICD-10) diagnosis of RA, independent of the beginning of symptoms. Data on rheumatoid factor (RF) tests and tests for antibodies to cyclic citrullinated peptides (anti-CCP) were retrieved from the databases of the two clinical immunology laboratories in the area. Patients with at least one positive test for RF or anti-CCP were considered positive for this marker.

All available reports on radiographs of hands and feet were reviewed. Based on the radiology reports, patients were classified as ever having arthritic erosions, typical of RA, or not. Data on smoking history, time of cessation, if applicable, number of children given birth to, and ever use of hormonal replacement therapy (HRT) were collected.

### Hormonal exposure variables

Data on age at menopause, breastfeeding history, use of OC, and level of formal education were retrieved from the self-administered questionnaire used in the MDCS, and assessed on average 5.5 years before RA diagnosis. All female individuals in the present sample who answered the MDCS questionnaire were 45 years of age or older. If they reported menopause beginning after the age of 45, or answered that they had not yet reached menopause, they were classified in the group "normal/late menopause." If they had reached menopause before the age of 46, they were included in the group "early menopause."

Breastfeeding history was stratified into never having breastfed, cumulative breastfeeding of 1 to 12 months, or cumulative breastfeeding of ≥ 13 months, as previously described [2]. Based on information on the use of OC, cases were divided into three groups; never-users, 1 to 5 years of use, and > 5 years of use. Nonresponders were excluded from the analyses.

## Statistics

Clusters of RA severity were identified by using SPSS TwoStep Clustering algorithm (version 19). This algorithm allows the use of both categoric and continuous variables in the same model. Selected outcome measures were based on data availability and clinical plausibility: biologic treatment, presence of radiographic erosions, RF status, and disability 5 years after diagnosis, measured by using the HAQ. The HAQ score at 5 years was included in the model because data were available in a greater number of patients at this time point. These outcome measures were added to the algorithm, and different numbers of natural groupings were explored.

The algorithm initially identified six clusters, but these were considered difficult to interpret from a clinical standpoint, and the small sample for each cluster was a concern. In the subsequent analysis, the algorithm was pre-set to identify three clusters. The model identifying three clusters was chosen because these clusters were considered to reflect clinically relevant phenotypes. Only after this decision had been made was the distribution of such clusters by baseline hormonal exposures examined by using the χ^2 ^test.

For the individual outcome parameters, only descriptive data without formal statistical comparisons were presented. Analysis of covariance (ANCOVA) was used to compare mean HAQ between subsets defined by hormonal predictors, adjusted for age.

The regional research ethics committee for southern Sweden approved the study. All patients gave their informed consent to be included in the Malmö RA registers and the MDCS database. No informed consent was obtained specifically for the present study.

## Results

Of the 136 incident female patients previously described [2], two patients on long-term follow-up were found to develop a phenotype compatible with erosive osteoarthritis rather than RA, and were therefore excluded from this study. Median time from inclusion in MDCS to RA diagnosis was 5.5 years. The mean age at RA diagnosis was 63.4 years, and the mean disease duration at the last follow-up was 10.1 years (SD, 3.5). Ninety-four (71.2%) were RF positive. Twenty-eight percent received biologic treatment during follow-up, and the median number of DMARDs used was two. Almost 60% of the patients had documented radiographic erosions, and 5% had severe extraarticular manifestations (Table 1). Availability of data on HAQ was limited and varied for different time points (Table 1), but no major differences was noted in the proportions with missing HAQ at different time points between those with early and normal/late menopause (52% versus 46% at diagnosis, 52% versus 36% at 1 year, 24% versus 33% at 5 years, and 52% versus 52% at 10 years).

**Table 1:** Patient characteristics

	All patients *n *= 134
Mean age at diagnosis; years (SD) (range)	63.2 (8.3) (47-80)

Mean follow-up time; years (SD)	10.1 (3.5)

RF positive (%)	94/132 (71.2)

Anti-CCP positive (%)	34/57 (59.6)

Smoking at diagnosis (%)	39/118 (33.1)

Low formal education (< 8 years of school) (%)	66/128 (49.3)

Prednisolone at diagnosis (%)	39/120 (32.5)

Biological treatment ever (%)	37/133 (27.8)

Number of used DMARDS; median (IQR)	2 (1-3)

> 3 DMARDs used (%)	30 (22.4)

DMARDS in combination (%)	54 (40.3)

Documented radiographic erosions (ever) (%)	71/125 (56.8)

Severe extraarticular manifestations (%)	7 (5.2)

Median HAQ at diagnosis^a^	0.87

Median HAQ 1 year after diagnosis^a^	0.75

Median HAQ 5 years after diagnosis^a^	0.75

Median HAQ 10 years after diagnosis^a^	0.88

CCP, cyclic citrullinated peptide; DMARD, disease-modifying antirheumatic drug; HAQ, Health Assessment Questionnaire; IQR, interquartile range; RF, rheumatoid factor; SD, standard deviation. ^a^Data available for HAQ at diagnosis in 70 cases, for HAQ at 1 year in 82 cases, for HAQ at 5 years in 92 cases, and for HAQ at 10 years in 63 cases.

All patients with complete data for the relevant outcome variables (*n *= 90) were included in the cluster analysis. Three different clusters of RA severity were identified, of which cluster I represented a severe RA phenotype; cluster II, a moderate/mild RF-positive RA phenotype; and cluster III, a moderate/mild RF-negative RA phenotype (Table 2).

**Table 2:** Distribution of clusters of clinical outcomes

	Cluster I: Severe RA (*n *= 26)	Cluster II: Mild/moderate seropositive RA (*n *= 25)	Cluster 3: Mild/moderate seronegative RA (*n *= 39)
Treated with biologics (ever)	100%	0	0

RF positive	88.5%	100%	0

Documented erosions on radiographs (ever)	84.6%	56.4%	52.0%

HAQ after 5 years			
Mean (SD)	1.17 (0.61)	0.74 (0.61)	0.88 (0.66)
Median (IQR)	1.00 (0.72-1.66)	0.63 (0.38-0.88)	0.88 (0.25-1.25)

HAQ, Health Assessment Questionnaire; IQR, interquartile range; RF, rheumatoid factor; SD, standard deviation.

Because HAQ-score values had a normal distribution among patients in cluster I, and a skewed distribution in clusters II and III, means with standard deviations as well as medians with interquartile ranges are presented (Table 2). In the group with severe RA, all patients had been treated with biologics; 89% were RF positive, 85% had documented erosions, and the mean HAQ score after 5 years was 1.17. In the mild/moderate RF-positive and RF-negative clusters, no one had received biologics; the proportions with documented erosions was lower, and mean HAQ scores after 5 years were lower than those in the severe RA cluster (Table 2).

### Hormonal factors and disease outcome

**Figure 1:**
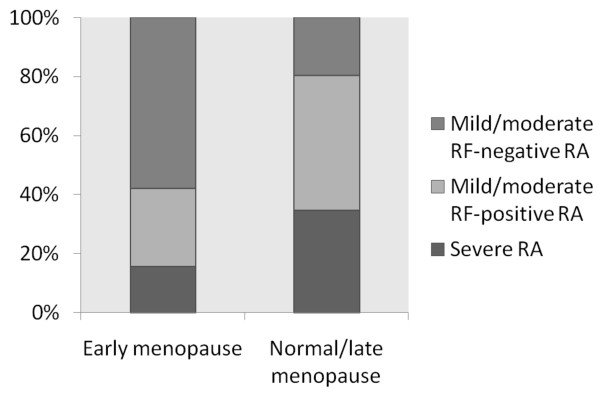
**Distribution of clusters of clinical outcomes defining disease severity by age at menopause**. *P *= 0.005.

The duration of follow-up was similar in patients with a history of early versus normal/late menopause (Table 3) and also similar across strata of length of breastfeeding (Table 4) and use of OC (Table 5). No major difference was found in the mean age at diagnosis between patients with early menopause (65.2 years) and normal/late menopause (62.8 years). A significant difference appeared in the distribution of patients between the clusters, depending on menopausal age (*P *= 0.005), with more women with early menopause developing RF-negative mild/moderate RA (Figure 1). The age-adjusted mean HAQ scores were lower among women with early menopause at all time points (Table 3). No significant interaction occurred between age at RA diagnosis and early versus normal/late menopause status in analyses of their impact on HAQ (data not shown). In analyses adjusted for age, a borderline association was found between early menopause and lower HAQ at diagnosis (β = -0.38; 95% CI, -0. 78 to 0.01), with a similar tendency at other time points (data not shown).

**Table 3:** Clinical outcomes by age at menopause

	Early menopause **(< 46 years) (*n *= 25)**^a^	Normal/late menopause **(≥ 46 years) (*n *= 102)**^a^
Mean age at diagnosis; years (SD)	65.2 (6.94)	62.8 (8.48)

Mean follow-up time; years (SD)	10.5 (3.0)	10.1 (3.6)

> 3 DMARDs used (%)	5/25 (20.0)	23/101 (22.8)

DMARDs in combination (%)	8/25 (32.0)	45/101 (44.6)

Biological treatment ever (%)	3/25 (12.0)	33/101 (32.7)

Documented radiographic erosions (ever) (%)	14/23 (60.9)	52/96 (54.2)

RF positive (%)	11/25 (44.0)	78/100 (78.0)

Mean HAQ at diagnosis (CI)^b^	0.58 (0.22-0.93) (*n *= 12)	0.96 (0.79-1.12) (*n *= 55)

Mean HAQ 1 year after diagnosis (CI)^b^	0.45 (0.08-0.83) (*n *= 12)	0.86 (0.70-1.02) (*n *= 65)

Mean HAQ 5 years after diagnosis (CI)^b^	0.88 (0.60-1.15) (*n *= 19)	0.90 (0.76-1.05) (*n *= 68)

Mean HAQ 10 years after diagnosis (CI)^b^	0.67 (0.34-1.00) (*n *= 12)	0.98 (0.82-1.14) (*n *= 49)

Cluster distribution (*P *= 0.005)		
Severe RA (%)	3 (15.8%)	23 (34.8%)
Mild/moderate seropos RA (%)	5 (26.3%)	30 (45.5%)
Mild/moderate seroneg RA (%)	11 (57.9%)	13 (19.7%)

CI, confidence Interval; HAQ, Health Assessment Questionnaire; RF, rheumatoid factor; SD, standard deviation. ^a^In case of missing data, numbers (*n*) indicated for each variable. ^b^Adjusted for age at diagnosis.

**Table 4:** Clinical outcomes by duration of breastfeeding

	Breastfeeding 0 months **(*n *= 43)**^a^	Breastfeeding 1-12 months **(*n *= 72)**^a^	Breastfeeding > 12 months **(*n *= 19)**^a^
Mean age at diagnosis; years (SD)	63.6 (8.37)	62.8 (8.30)	64.2 (8.27)

Mean follow-up time; years (SD)	9.6 (3.4)	10.3 (3.7)	10.5 (2.8)

> 3 DMARDs used (%)	8/43(18.6)	17/71 (23.9)	5/19 (26.3)

DMARDs in combination (%)	14/43 (32.6)	32/71 (45.1)	8/19 (42.1)

Biological treatment ever (%)	10/43(23.3)	21/71 (29.6)	6/19 (31.6)

Documented radiographic erosions (ever) (%)	25/39 (64.1)	36/70 (51.4)	10/16 (62.5)

RF positive (%)	28/43 (65.1)	53/71 (74.6)	13/18 (72.2)

Mean HAQ at diagnosis (CI)^b^	0.91 (0.64-1.18) (*n *= 21)	0.89 (0.69-1.09) (*n *= 39)	0.86 (0.47-1.25) (*n *= 10)

Mean HAQ 1 year after diagnosis (CI)^b^	0.76 (0.49-1.03) (*n *= 24)	0.86 (0.66-1.06) (*n *= 44)	0.68 (0.32-1.03) (*n *= 14)

Mean HAQ 5 years after diagnosis (CI)^b^	0.91 (0.68-1.14) (*n *= 31)	0.90 (0.72-1.09) (*n *= 49)	0.85 (0.48-1.22) (*n *= 12)

Mean HAQ 10 years after diagnosis (CI)^b^	0.78 (0.50-1.06) (*n *= 16)	0.94 (0.76-1.13) (*n *= 38)	1.08 (0.70-1.45) (*n *= 9)

Cluster distribution (*P *= 0.88)			
Severe RA (%)	8/31 (25.8)	15/48 (31.2)	3/11 (27.3)
Mild/moderate seropos RA (%)	13/31 (41.9)	20/48 (41.7)	6/11 (54.5)
Mild/moderate seroneg RA (%)	10/31 (32.3)	13/48 (27.1)	2/11 (18.2)

CI, confidence interval; HAQ, Health Assessment Questionnaire; RF, rheumatoid factor; SD, standard deviation. ^a^In case of missing data, numbers (n) indicated for each variable. ^b^Adjusted for age at diagnosis.

**Table 5:** Clinical outcomes by history of use of oral contraceptives

	**Never used (*n *= 67)**^a^	**1-5 years of use (*n *= 17)**^a^	**> 5 years of use (*n *= 43)**^a^
Mean age at diagnosis; years (SD)	66.2 (7.82)	58.8 (8.19)	60.1 (7.17)

Mean follow-up time; years (SD)	10.4 (3.3)	9.9 (3.1)	10.0 (3.8)

> 3 DMARDs used (%)	11/67 (16.4)	4/17 (23.5)	14/42 (33.3)

DMARDs in combination (%)	26/67 (38.8)	8/17 (47.1)	20/42 (47.6)

Biological treatment ever (%)	14/67 (20.9)	4/17 (23.5)	19/42 (45.2)

Documented radiographic erosions (ever) (%)	37/62 (59.7)	10/17 (58.8)	20/40 (50.0)

RF positive (%)	46/66 (69.7)	10/16 (62.5)	34/43 (79.1)

Mean HAQ at diagnosis (CI)^b^	0.75 (0.53-0.97) (*n *= 34)	0.93 (0.42-1.43) (*n *= 6)	1.06 (0.81-1.30) (*n *= 27)

Mean HAQ 1 year after diagnosis (CI)^b^	0.83 (0.61-1.05) (*n *= 39)	0.64 (0.20-1.09) (*n *= 9)	0.81 (0.56-1.06) (*n *= 29)

Mean HAQ 5 years after diagnosis (CI)^b^	0.93 (0.75-1.11) (*n *= 47)	0.80 (0.46-1.15) (*n *= 13)	0.86 (0.62-1.10) (*n *= 26)

Mean HAQ 10 years after diagnosis (CI)^b^	0.79 (0.59-1.00) (*n *= 33)	1.20 (0.77-1.63) (*n *= 7)	1.00 (0.75-1.26) (*n *= 21)

Cluster distribution (*P *= 0.56)			
Severe RA (%)	12/47 (25.5)	3/12 (25.0)	11/25 (44.0)
Mild/moderate seropos RA (%)	21/47 (44.7)	5/12 (41.7)	9/25 (36.0)
Mild/moderate seroneg RA (%)	14/47 (29.8)	4/12 (33.3)	5/25 (20.0)

CI, confidence interval; HAQ, Health Assessment Questionnaire; RF, rheumatoid factor; SD, standard deviation. ^a^In case of missing data, numbers (*n*) indicated for each variable. ^b^Adjusted for age at diagnosis.

No significant differences in the distribution of clusters of clinical-outcome measures were seen in analysis by breastfeeding history (*P *= 0.88) or previous use of OC (*P *= 0.56) (Tables 4 and 5). Women who had used OC > 5 years tended to be in the cluster of severe RA, and the mean age at RA diagnosis was lower in this subset (Table 5). Overall, individuals who were younger at diagnosis were more likely to be treated with biologics (data not shown). No major differences were noted in age at diagnosis, depending on breastfeeding history (Table 4).

Women who hade normal/late menopause were more likely to receive treatment with biologics and DMARDS in combination, but no major differences appeared in the number of different DMARDS used during the follow-up (Table 3).

## Discussion

We previously showed that hormonal factors such as early menopause and breastfeeding history influence the risk of RA [2,14]. In particular, early menopause was a robust risk factor for RA [14]. In this study, we demonstrated that early menopause predicts a milder type of RA.

Estrogen is suggested to suppress cellular immunity directly but to stimulate the humoral immune system [21]. A decrease in estrogen levels may thus contribute to a T-cell differentiation skewed toward the T-helper 1 (Th1) subset. This has previously been implicated as an important part of the pathogenesis of RA [22,23]. Further studies should examine the impact of the timing of menopause on immunologic and inflammatory pathways leading to RA.

Dysfunctions of the hypothalamic-pituitary-adrenocortical axis (HPA axis), the hypothalamic-pituitary-gonadal (HPG axis), and the autonomic nervous system have all been suggested to be a part of the complex pathogenesis of RA [24,25]. Similar observations have been made in primary Sjögren syndrome [26]. A gradual decline in the function of the HPG axis is considered a key element in the development of menopause [27,28]. We propose that women with a less-responsive HPG axis, leading to an increased risk of early menopause, may also have a primarily malfunctioning or "sluggish" HPA axis. A reduced response of the HPA axis to inflammation would make them more susceptible to develop a chronic inflammatory disease. In support of this, it has been found that women who develop RA later in life have higher birth weight than do normal controls [29,30], and some data link high birth weight with a less-responsive HPA function later in life [31]. This may reflect an impact of HPA function on chronic inflammation in general, rather than on the specific pathogenesis of erosive arthritis.

In this study, women with a history of early menopause in whom RA developed were more likely to have a mild disease course, and less likely to progress to severe RA. This suggests that different predictors may be associated with distinct clinical outcomes. For example, other known risk factors for RA, such as the shared epitope of HLA-DRB1, and smoking, may, in comparison with early menopause, be more specifically associated with a severe phenotype of RA [32,33].

A recent study [12] suggested that OC use is associated with mild RA and demonstrated a reduction of disability, measured by HAQ over time, in former OC users, compared with nonusers. Furthermore, current OC users had lower HAQ scores at baseline and over time than did previous users. Jorgensen *et al. *[13] found that OC is both protective for RA and associated with a mild phenotype in individuals who develop RA. Yet another study did not find a protective effect of current use of OC for RA [34]. Because our patients were older, with a mean age at diagnosis of 63 years (range, 47 to 80 years), only one patient reported current use of OC at diagnosis, and we could therefore not address the effect of current use of OC. The mean time between OC use and RA diagnosis was 20 years, which may explain the limited impact of former OC use on the severity of RA. In our sample, the former users rather tended to develop a more-severe RA, at least by some outcome measures. This could be biased by their younger age at diagnosis. In particular, the difference in the proportion treated with biologics may be due to more extensive prescription of such drugs for younger individuals with RA, as was the case in the present study and also reported in a recent national survey [35].

Barrett *et al. *[36] suggested that breastfeeding is a risk factor for a more-severe inflammatory polyarthritis in a small group of genetically susceptible women, based on the linkage between HLA-DRB1 alleles and the prolactin gene on chromosome 6 [37]. In a prospective short-term study of pregnant women with inflammatory polyarthritis, they found that women who breastfed after a first pregnancy had a more-severe disease 6 months postpartum compared with non-breastfeeders and previous breastfeeders [36]. However, in the present study, we could not demonstrate any significant long-term effect of breastfeeding on the severity of RA. Our study design excludes the smaller group of women who develop RA during their younger years, which could affect our results. The immune-stimulating effect of prolactin could also be short term, and breastfeeding could have a different long-term immune-modulating effect, suggested by the results from the Nurses Health Study [38] and our previous results [2].

Limitations of this study include the retrospective data collection, because information in medical records is sometimes incomplete and not always coherent, due to diverse assessments by different physicians. Because risk-factor information and clinical records are two completely unrelated sources of information, it is unlikely that such incompleteness is differential between risk factor-exposure categories. Nevertheless, the relatively large proportion of missing data of HAQ at specific time points is a limitation. Because some physicians may be less prone to collect HAQ routinely, and this may also change over time, we cannot exclude a systematic bias based on this. However, no major differences were found in the proportions with missing HAQ between those with early and normal/late menopause.

Because of differences in management practice, the methods used to define clusters of disease severity may not be valid in other populations. Another limitation is the relatively small sample size, which affects analyses of subsets with different risk-factor profiles.

Strengths include the prospective assessment of hormone-related factors before disease onset, which limits the risk of recall bias, and the population-based approach, which reduces the risk of selection bias.

## Conclusions

We report that early menopause is associated with a mild type of RA among women with disease onset after 45 years of age. Hormonal changes may influence pathways that are distinct from those leading to severe, progressive disease. To our knowledge, this is the first study on the impact of menopausal age on the severity of RA, and our findings must be confirmed in other studies of larger samples. Such studies require that data on the exposure (that is, age at menopause) have been collected in a consistent and valid manner. If our results are confirmed, it may in the future be relevant to check for early menopause in women with RA.

## Key messages

• Early menopause is associated with a mild seronegative RA phenotype.

• Breastfeeding history and former use of oral contraceptives did not influence the severity of RA.

• Hormonal changes may influence pathways leading to a mild type of rheumatoid arthritis.

## Abbreviations

ACR: American College of Rheumatology; anti-CCP: antibodies to cyclic citrullinated peptides; ANCOVA: analysis of covariance; DMARDs: disease-modifying antirheumatic drugs; HAQ: Health Assessment Questionnaire; HRT: hormone replacement therapy; HPA axis: hypothalamic-pituitary- adrenocortical axis; HPG axis: hypothalamic-pituitary-gonadal axis; MDCS: Malmö Diet and Cancer Study; OC: oral contraceptive; RA: rheumatoid arthritis; RF: rheumatoid factor; SSATG: South Swedish Arthritis Treatment Group; Th-1 cell: T-helper 1 cell.

## Competing interests

The authors have no potential conflicts of interest regarding this article.

## Authors' contributions

MP carried out data collection, participated in the design of the study and the statistical analysis, and wrote the article. J-ÅN contributed to the statistical analysis and writing. UB identified cases and controls and contributed to the writing of the article. LJ participated in the design of the study and writing. CT contributed to the study design, statistical analysis, research supervision, and writing. All authors read and approved the final version.
